# Making microbiology of the built environment relevant to design

**DOI:** 10.1186/s40168-016-0152-7

**Published:** 2016-02-16

**Authors:** G. Z. Brown, Jeff Kline, Gwynne Mhuireach, Dale Northcutt, Jason Stenson

**Affiliations:** Energy Studies in Buildings Laboratory, Department of Architecture, University of Oregon, Eugene, OR USA; Biology and the Built Environment Center, University of Oregon, Eugene, OR USA; Department of Landscape Architecture, University of Oregon, Eugene, OR USA

**Keywords:** Architecture, Buildings, Microbiome, Microbial ecology, Bioinformed design, Sustainability

## Abstract

Architects are enthusiastic about “bioinformed design” as occupant well-being is a primary measure of architectural success. However, architects are also under mounting pressure to create more sustainable buildings. Scientists have a critical opportunity to make the emerging field of microbiology of the built environment more relevant and applicable to real-world design problems by addressing health and sustainability in tandem. Practice-based research, which complements evidence-based design, represents a promising approach to advancing knowledge of the indoor microbiome and translating it to architectural practice.

## Background

Prior to the widespread adoption of vaccines and antibiotics, good building design was considered as an important factor in maintaining health [1]. The perception of buildings as “health machines” significantly influenced Modernist architects such as Le Corbusier and Tony Garnier, who designed buildings to admit sunlight and fresh air due to concern for occupant health [2]. Today, chronic and autoimmune disorders are escalating [3], and inadequate exposure to microbial diversity during early childhood is thought to play a role [4]. We know that architectural choices such as ventilation type influence indoor microbial communities [5], so perhaps the time is ripe to again regard quality architecture as a public health service. However, to design “bioinformed” buildings that foster well-being [6], architects need scientific knowledge that addresses the conditions and constraints of their work. Microbiology of the built environment (MoBE) research represents a prime opportunity for such design-science collaboration.

Architectural design is poised to undergo a revolution over the next few decades in response to climate change, urbanization, and population growth. Climate change is a threat to our way of living. Because building energy use contributes over 40 % of total global carbon emissions [7], many architects have pledged to achieve net zero energy use for all new buildings by 2030. At the same time, over 50 billion square feet of residential and commercial buildings are projected to be constructed by 2040 in the USA alone [8], to accommodate urbanization and population growth. These buildings will have an average lifespan of 50–100 years. Together, these trends suggest that if MoBE researchers want to influence the definition of healthy and sustainable buildings for the next century, now is the time to act.

## Main text

Unfortunately, scientific research can fail to inform architectural practice. First, research results may not reach practitioners. Scientific knowledge is commonly disseminated in peer-reviewed journals and academic conferences, yet architects typically gain professional knowledge from other sources, including trade shows, magazines, and continuing education workshops. Second, the research may not address questions that seem important or relevant to architects. Finally, researchers can fail to synthesize their findings into design tools or guidelines. We believe that these barriers can be overcome with greater collaboration between architects and MoBE scientists on both research and design projects. In particular, architects’ knowledge can inform research questions, leading to timely investigations that are pertinent to building design.

Evidence-based design, a descendant of evidence-based medicine, is the use of best available scientific knowledge as a basis for design decisions and has become popular in healthcare architecture [9]. However, the results obtained in controlled research environments may not be realized under real-world conditions, since each building is unique in its site context, design characteristics, operation, and occupancy. Following the recent trend in clinical healthcare for practice-based research [10], we contend that MoBE theory can be developed and tested through interventions in real practice, in addition to conventional research methods. Practice-based research would complement evidence-based design, leading to a positive feedback loop where research influences design and vice versa.

An example of this approach might be microbiome “experiments”—design changes that alter the microbial community of a building while staying within architectural best practices—implemented during the design phase of new or retrofit buildings. Given the pressing need for energy-efficient design, these studies should focus on low-energy design strategies, such as daylighting and natural ventilation. An example of such a study occurred at the University of Oregon, where a mixed-use building was designed so that half of the offices used operable windows to provide ventilation while the other half used a conventional mechanical system. Dust samples from the offices showed clear differences in the microbial communities that were primarily explained by the source of ventilation air [11]. Another example is the Bullitt Center in Seattle, WA, which is the only office building to have attained Living Building Challenge certification. Designed to improve occupant health based on best available knowledge, this building is currently being used to investigate relationships among design, occupant health, and microbial dynamics [12].

## Conclusions

MoBE is a critical juncture of study because humans spend most of their time inside buildings, and the microorganisms encountered there can impact public health. One might object that the current level of MoBE knowledge is inadequate to purposefully design healthier and more sustainable buildings. But we would argue that architectural design changes based on poorly understood microbe-human dynamics are the norm. We believe the following actions are necessary to further advance the MoBE field:

- Implement a new model of practice-based research where new and retrofit construction projects are considered as study vehicles to test microbiome theories.
- Cross-train next generation design scientists who can be healthy building consultants.
- Use alternative dissemination outlets to reach architectural practitioners, including trade shows, workshops, and design competitions.

In closing, we reaffirm that architects and other designers are committed to improving occupant health through strategies such as bioinformed design. However, in order to be applied in building projects, scientific knowledge must address real-world constraints and be translated into formats that are accessible to design practitioners.

## Abbreviations

MoBE: microbiology of the built environment
